# A new classification of periampullary diverticulum: cannulation of papilla on the inner margins of the diverticulum (Type IIa) is more challenging

**DOI:** 10.1186/s12876-023-02862-9

**Published:** 2023-07-25

**Authors:** He-xian Shi, Yong-qiang Ye, Hai-wang Zhao, De-cai Kong, Shan-zhou Huang, Qian Yan, Yu-bin Chen, Ping Zhang, Sheng Chen, Bao-hua Hou, Chuan-zhao Zhang

**Affiliations:** 1 The Second School of Clinical Medicine, Southern Medical University, Guangzhou, 510515 China; 2 Department of General Surgery, Guangdong Provincial People’s Hospital (Guangdong Academy of Medical Sciences), Southern Medical University, Guangzhou, 510080 China; 3 Department of Hepatobiliary Surgery, HeZe Municipal Hospital, HeZe, 274000 Shandong China; 4 Department of Gastrointestinal Surgery, HeZe Municipal Hospital, HeZe, 274000 Shandong China; 5 School of Medicine, South China University of Technology, Guangzhou, 51000 China

**Keywords:** Periampullary diverticula, Cannulation, Post-ERCP pancreatitis, Perforation

## Abstract

**Background:**

Periampullary diverticulum (PAD) may make the performance of endoscopic retrograde cholangiopancreatography (ERCP) in patients with choledocholithiasis more difficult and may increase complication rates. The present study evaluated the effects of PAD on first-time ERCP in patients with choledocholithiasis.

**Methods:**

Outcomes were compared in patients with and without PAD and in those with four types of PAD: papilla located completely inside the diverticulum (type I), papilla located in the inner (type II a) and outer (type II b) margins of the diverticulum; and papilla located outside the diverticulum (type III).

Parameters compared included cannulation time and rates of difficult cannulation, post-ERCP pancreatitis (PEP) and perforation.

**Results:**

The median cannulation times in patients with types I, II a, II b, III PAD and in those without PAD were 2.0 min, 5.0 min, 0.67 min, 3.5 min, and 3.5 min, respectively, with difficult cannulation rates in these groups of 7.4%, 31.4%, 8.3%, 18.9%, and 23.2%, respectively*.* The rates of PEP in patients with and without PAD were 5.3% and 5.1%, respectively. Four patients with and one without PAD experienced perforation.

**Conclusions:**

The division of PAD into four types may be more appropriate than the traditional division into three types. Cannulation of type I and II b PAD was easier than cannulation of patients without PAD, whereas cannulation of type II a PAD was more challenging. PAD may not increase the rates of PEP.

## Introduction

The incidence of periampullary diverticulum (PAD) has been reported to range from 5.6% to 46.1% [1–3]. Based on their relationship with papilla, PAD has been traditionally divided into three types: papilla located inside the PAD (type I); papilla located in the margins of the PAD (type II); and papilla located close to the PAD (type III) [4, 5]. Although PAD does not cause clinical symptoms in ordinary circumstances, it is thought to be related to choledocholithiasis and to affect the performance and results of endoscopic retrograde cholangiopancreatography (ERCP). Many have assessed the effects of PAD on ERCP, but it is unclear whether PAD increases operational difficulties and complications of ERCP [1, 2, 6–11]. In addition, most studies have compared patients with and without diverticulum, with few studies to date assessing the effects and complications of ERCP in patients with different types of PAD.

To evaluate the effects of PAD in patients undergoing ERCP, patients were retrospectively divided into those with and without PAD. In addition, patients with PAD were subdivided into four types, those with papilla located completely inside the diverticulum (type I); papilla located in the inner (type II a) and outer (type II b) margins of the diverticulum; and papilla located outside the diverticulum (type III). The comparison between our division method and traditional division is shown in Table 1. Outcomes were compared, including cannulation times and rates of difficult cannulation, post-ERCP pancreatitis (PEP) and perforation.

**Table 1:** Comparison between our classification method and traditional classification

location	completely inside	inner margin	outer margin	outside
new	type I	type II a	type II b	type III
traditional	type I	type II		type III

## Methods

This study included 386 inpatients with common bile duct stones who were hospitalized and underwent ERCP for the first time from 1 May 2018 to 31 December 2021, all the procedures were completed by two experts who had performed more than 500 operations. Patients with a surgically altered anatomy and those who underwent more than one session of ERCP were excluded.

**Fig. 1:**
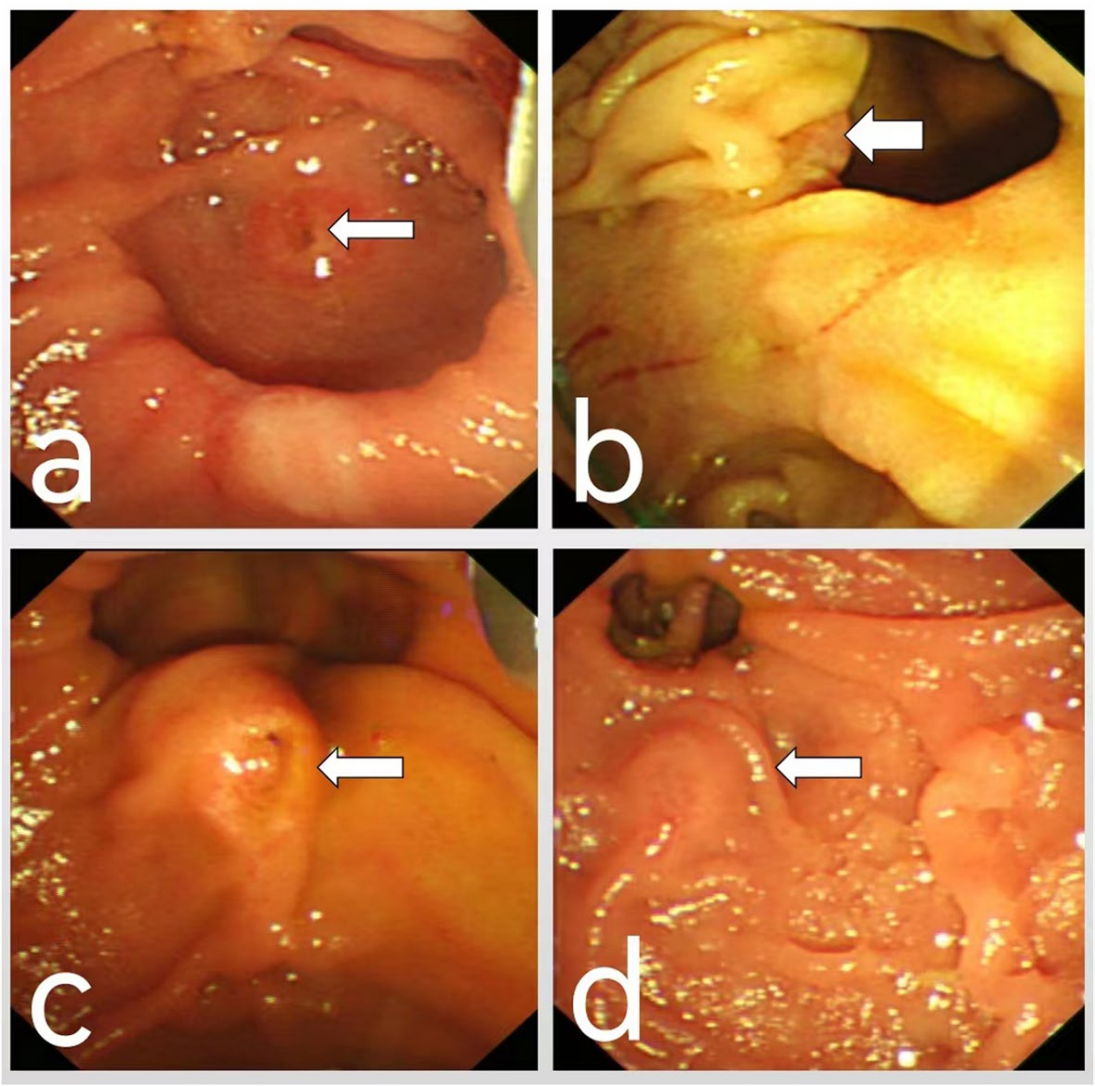
Illustrations of the four subtypes of PAD. **a** Type I, **b** Type II a, **c** Type II b, **d** Type III. The definitions of the four subtypes are described in the text

Patients who met the inclusion criteria were divided into two groups, those with PAD (*n* = 209) and those without PAD (*n* = 177). Patients with PAD were further subdivided into four types according to the relationship between the diverticulum and the papilla (Fig. 1): those with papilla located inside the diverticulum, not adjacent to the margins (type I, *n* = 27 patients), papilla located in the inner (type II a, *n* = 35) and outer (type II b, *n* = 36) margins of the diverticulum, and papilla located outside the diverticulum (type III, *n* = 111). Parameters compared in patients with and without PAD included age, gender, cannulation time, and rates of difficult cannulation, PEP and perforation after ERCP. Parameters compared in patients with the four types of PAD and in patients without PAD included cannulation time and difficult cannulation rate.

The study protocol conformed to the ethical guidelines of the 1975 Declaration of Helsinki (6th revision, 2008).

The start of cannulation was defined as the time that the sphincterotome was pushed out from the endoscope [12]. Successful cannulation was defined as extraction of bile or cannula well inside the common bile duct (CBD), as determined by X-ray identification of the guide wire [13]. Difficult cannulation was defined as the inability to achieve selective biliary cannulation by standard ERCP techniques within 10 min or up to five cannulation attempts or failure of access to the major papilla [14]. Plasma amylase concentrations were measured 4–6 h after ERCP and the following morning. PEP was defined as “clinical pancreatitis with amylase at least three times the upper limit of normal more than 24 h after the procedure, requiring hospital admission or a prolongation of planned admission” [15].

Categorical variables of age, PEP and perforation were compared by Pearson’s chi-square tests, cannulation time and difficult cannulation ratio were compared by Nonparametric Wilcoxon Rank-Sum Test. All statistical analyses were performed using SPSS, version 22 software, with *P* values < 0.05 considered statistically significant.

## Results

### Study population

A total of 386 patients were enrolled in this study, 209 (54.1%) in the PAD group and 177 (45.9%) in the non-PAD group. Mean overall patient age was 70.1 years, with patients in the PAD group being significantly older than those in the non-PAD group (71.92±11.76 years versus 68.03±13.58 years, *P*=0.003). The incidence of PAD was significantly lower in patients aged <70 years than in those aged ≥70 years (44.3% [70/158] versus and 61.0% [139/228], *P*=0.001) and was 46.8% (36/77) in patients aged 60–70 years (Table 2). Of the 209 patients in the PAD group, 27 (12.9%) were divided as type I , 35 (16.7%) as Type II a, 36 (17.2%) as Type II b, and 111 (53.1%) as type III. There were no significant differences in sex, cannulation time, difficult cannulation rate or complications of PEP and perforation between patients with and without PAD (Table3).

**Table 2:** Rates of diverticulum in patients < 70 and ≥ 70 years

Age, years	PAD	Non-PAD	Total
< 70	70	88	158
≧70	139	89	228
	209	177	386

**Table 3:** Baseline characteristics and outcomes of ERCP in patients with and without PAD

Clinical characteristics	PAD Group (*n* = 209)	Non-PAD Group (*n* = 177)	*P* value
Mean age, years	71.92 ± 11.76	68.03 ± 13.58	0.003
Male	53.1%	52.5%	0.911
Female	46.9%	47.5%	
Cannulation time, min	2.50 (1.00, 6.00)	3.50 (1.00, 7.75)	0.183
Difficult cannulation	17.7%	23.2%	0.183
PEP	5.3%	5.1%	0.937
Perforation	1.9%	0.6%	0.474

### Biliary cannulation

All 386 patients were successfully cannulated. The median cannulation time did not differ in patients with and without PAD (2.50 min [range, 1.00–6.00 min] versus 3.50 min [range, 1.00–7.75 min], *P* = 0.183), but differed markedly in the four types of PAD (Table 4). Although cannulation time in patients with type III PAD did not differ from that of patients without PAD, it was much shorter in patients with type II b PAD (0.67 min [range, 0.50–2.88 min]) and longest in patients with type II a PAD. (5.00 min [range, 1.50–14.00 min]).

**Table 4:** Cannulation time and difficult cannulation rates in patients with and without PAD

	Non-PAD Group	PAD Group				*P* value
	B	Type I	Type IIa	Type IIb	Type III	
Cannulation time (min)	3.50 (1.00, 7.75)^a^	2.00(1.00, 3.00)^bc^	5.00 (1.50, 14.00)^ac^	0.67 (0.50, 2.88)^b^	3.50 (1.50, 7.50)^a^	< 0.001
Difficult cannulation	41 (23.2%)^ab^	2 (7.4%)^b^	11 (31.4%)^a^	3 (8.3%)^ab^	21(18.9%)^ab^	0.046

Difficult cannulation rates were similar in patients with and without PAD (17.7% [37/209] versus 23.2% [41/177], *P* = 0.183; Table 3o). Among patients with PAD, those with type I had the lowest difficult cannulation rate (7.4%), whereas those with type II a had the highest (31.4%) (Table 4). Difficult cannulation rates did not differ significantly among patients with types II b and IIIPAD and patients without PAD (Table 4).

### PEP and perforation

The incidence of PEP was similar in patients with and without PAD (5.3% [11/209] versus 5.1% [9/177], *P* = 0.937).

Only one patient (0.6%) in the non-PAD group experienced type III perforation(duodenal perforation), which occurred during a prolonged operation lasting more than 100 min due to multiple huge stones in the CBD. Of the patients in the PAD group, one experienced type III perforation (bile duct perforation), and three experienced type IV perforation ( a small amount of gas was accumulated in the retroperitoneum during CT examination) (*P* = 0.474). The patient with type III perforation had a very hard stone in the CBD, with the basket damaged during repeated mechanical lithotripsy. Following stone extraction, this perforation was successfully blocked with a covered metal stent. Of the three patients with type IV perforation, they respectively had type II a II b, and III PAD. After receiving gastrointestinal decompression, antibiotic intravenous infusion, and parenteral nutritional support, they gradually recovered.

### Data among different groups under traditional classification

We also grouped all patients based on the traditional division of PAD(Tables 5 and 6), type I and III represent papilla inside and outside PAD, Type II represent papilla located inner and outer margin. Cannulation time, incidence of difficult cannulation, PEP, and perforation of each group were compared, and the results showed that there was no statistically significant difference in difficult cannulation,PEP and perforation incidence among the groups(P > 0.05). The only statistically significant difference found in the study was in the cannulation time of type I, which was significantly shorter than the other groups,. However, there were no significant differences in cannulation time between the other groups.

**Table 5:** Cannulation time and difficult cannulation comparation according traditional division

	Non-PAD Group	PAD Group			*P* value
		Type I	Type II	Type III	
Cannulation time (min)	3.50 (1.00, 7.75) b	2.00(1.00, 3.00) a	2.50(0.67,6.00) b	3.50 (1.50, 7.50) b	0.048
Difficult cannulation(%)	23.2%	7.4%	19.7%	18.9%	0.299

**Table 6:** PEP and perforation comparation according traditional division

		Non-PAD Group	PAD Group			*P* value
			Type I	Type II	Type III	
PEP	N	168(94.9%)	26(96.3%)	67(94.4%)	105(94.6%)	1.00
	Y	9(5.1%)	1(3.7%)	4(5.6%)	6(5.4%)	
perforation	N	176(99.4%)	27(100%)	69(97.2%)	109(98.2%)	0.379
	Y	1(0.6%)	0(0%)	2(2.8%)	2(1.8%)	

## Discussion

PAD is mainly found during CT or gastroscopy examinations. Its incidence ranges widely, from 5.6% (44/780) in Iran [1] to 46.1% (65/144) in South Korea [3]. The present study found that the incidence of PAD among patients undergoing ERCP was even higher, 54.1% (209/386). PAD is thought to increase with age, and the majority of the patients in this study were aged ≥ 70 years, which may account for the higher incidence of PAD in this population. Patients with PAD were significantly older than patients without PAD (71.92 ± 11.76 years vs. 68.03 ± 13.58 years; *p* < 0.05), with the incidence of PAD being significantly higher in patients aged ≥ 70 years than in those aged < 70 years (61.0% vs. 44.3%, *p* < 0.05). In contrast, the incidence of PAD was only 46.8% in patients aged 60–70 years. From our data, the incidence of diverticulum increased significantly from the age of 70, so we set up 70 years old as the cut-off point.

Based on the location of the papilla, PAD is usually divided into three types [4, 5]: papilla located inside the diverticulum (Type I), in the margins of the diverticulum (Type II), or outside the diverticulum (Type III); or into two types; papilla located inside or in the margins of the diverticulum (Type I) or near the diverticulum (Type II) [16, 17]. PAD has also been divided into four types, with two of these types, types II and IV, further divided into two subtypes each [18]. These division, however, are insufficient in determining the effects of different types of diverticulum on ERCP. When comparing the groups according to the traditional division, we found that there was no statistically significant difference in cannulation time between type II (papilla located in the margin of the diverticulum), type III (papilla located outside the diverticulum) and non-PAD groups. However, when we compared the groups according to the new classification, we found that cannulation of papilla located in the inner margin of the diverticulum was more difficult than cannulation of papilla located entirely within or in the outer margin of the diverticulum. Therefore, it is inappropriate to classify the diverticula when papilla located in the inner edge and those in the outer edge as the same type. In order to finely highlight the significant impact of various types of diverticula on cannulation, it is necessary to subdivide the diverticulum into two subtypes when papilla located in the inner and outer margin(type II a and type II b). Ultimately, we divided the diverticulum into three types, and type II was further subdivided into type II a and type II b. Individual diverticula in patients with two or more can also be divided into these types based on their positions. Type III diverticula is the most common, while type I diverticula is relatively rare.

Studies assessing the impact of PAD on cannulation have yielded conflicting results. Although the appearance of the diverticulum was thought to increase difficulties in cannulation and complications [1, 7, 19, 20], other studies have conflictingly found that the diverticulum did not affect cannulation time, made it easier, or increased overall complication rates [9, 16, 21, 22]. At present, only age differed in patients with and without PAD, with no significant differences in sex, cannulation difficulty, pancreatitis and perforation rates. In general, diverticula did not appear to make the ERCP process more difficult or increase the incidence of complications. However, comparisons of the non-PAD group with the different PAD subgroups yielded different results. For example, cannulation times did not differ significantly between patients with type III PAD and the non-PAD group, perhaps because the diverticulum was far removed from the papilla and did not affect the opening or direction of the papilla.

The cannulation time of Type II b PAD was significantly shorter than the times of the three other PAD subgroups, with entry of the guide wire into the bile duct being easier. Papilla located completely within the diverticulum (type I), were often easier to find and their openings were more obvious. Cannulation of this type was not as difficult as we thought it would be, taking less time than normal papillae. Also unexpectedly, cannulation of papilla in the inner margin of the diverticulum (Type II a) took the longest time. Approaching the papilla required repeated efforts to adjust the direction and length of the endoscope. Alternatively, other methods were needed to expose the papilla, making it easier to cannulate, which may have increased cannulation times.

Analysis of the incidence of the ERCP complications PEP and perforation found no differences between the PAD and non-PAD groups, a finding consistent with previous results [8–11]. In contrast, other studies have reported that complication rates are significantly higher in PAD than in non-PAD groups [1, 2, 6, 7, 20]. Factors associated with the occurrence of PEP include age, sex, bile duct diameter and the operation process [15].

The present study found that the occurrence of diverticulum did not increase the incidence of pancreatitis, possibly because diverticulum did not affect the cannulation and operation processes. One patient in the non-PAD group experienced perforation due to prolonged operation time, and one in the type III PAD group experienced perforation due to a hard stone. Another patient in the type III PAD group experienced postoperative perforation for unknown reasons, as did one patient each in the type II b and type II a PAD groups. None of the patients with type I PAD experienced perforation, but this does not mean that the operation was safe, because this group included the smallest number of patients and required a more careful operation. The walls of the diverticulum are thinner than normal duodenal walls, and are more likely to be perforated during surgery, especially in the presence of large stones, which require a longer operation time. Therefore, special care should be taken when papillae are located within or in the margins of the diverticulum. Secondary ERCP extraction of stones should be considered to avoid perforation in patients with large stones. There were some limitations in this study. The total number of enrolled cases was relatively small and the proportion of type I diverticula was low. Second, this is a retrospective study. Therefore, prospective studies with large sample size are needed to validate our findings in the future. Because many risk factors are associated with PEP and perforation, it was difficult to determine whether these complications are related to the presence of diverticulum. Due to the small numbers of patients in these subgroups and the low incidence rates of PEP and perforation, these complications were not compared statistically in the four PAD subgroups. Large multi-center trials are needed to determine the associations of diverticulum with PEP and perforation.

In conclusion, the present study found that the incidence of diverticulum is significantly higher in patients aged ≥ 70 years than those aged < 70 years. Our findings suggest that it may be more appropriate to divide the diverticulum into four types**.** Cannulation of types I and II b PAD was easier than cannulation of non-PAD, whereas cannulation of type II a PAD was more challenging. Although the appearance of PAD may not increase the occurrence of PEP, operators should try to avoid perforation when papilla are located in the margins or inside the diverticulum. Further research and analysis may be needed to fully understand the differences between these types of diverticula and their associated risks and complications in ERCP operations.

## Data Availability

The data used to support the findings of this study are available from the corresponding authors upon request.

## Abbreviations

PAD: Periampullary diverticulum
ERCP: Endoscopic retrograde cholangiopancreatography
PEP: Post-ERCP pancreatitis
CBD: Common bile duct
